# Breeding *Tm-1*-based tomato rootstocks, resistant to tomato brown rugose fruit virus, to impede soil-mediated viral infections

**DOI:** 10.3389/fpls.2026.1815342

**Published:** 2026-04-23

**Authors:** Edan Rochsar, Elisheva Smith, Kfir Bandel, Chen Klap, Elena Bakelman, Amnon Koren, Dani Zamir, Aviv Dombrovsky

**Affiliations:** 1 Department of Plant Pathology and Weed Research, Agricultural Research Organization - Volcani Institute, Rishon Lezion, Israel; 2 The Institute of Plant Sciences and Genetics, Faculty of Agriculture, The Hebrew University of Jerusalem, Rehovot, Israel; 3 Hishtil Nurseries, Nehalim, Israel

**Keywords:** disease symptom severity, grafted plants, *Tobamovirus*, ToBRFV, viral disease management

## Abstract

**Introduction:**

The *Tobamovirus fructirugosum*, tomato brown rugose fruit virus (ToBRFV) is a mechanically transmitted, seed- and soil-borne virus causing severe damage to tomato crops worldwide. Under monoculture conditions, infected residues enable ToBRFV to persist in soil, initiating infectious foci in newly planted seedlings. These plants are then exposed to foliar mechanical inoculations, facilitating disease spread. Management strategies include the use of ToBRFV-resistant germplasms.

**Methods:**

We screened 52 wild and 45 cultivated tomato varieties for ToBRFV resistance using foliar inoculation, symptom scoring, and ELISA. Lines harboring the *Tm-1* resistance gene were used to generate resistant rootstocks and scions, which were challenged with ToBRFV. Grafting combinations of resistant and susceptible plants were evaluated under controlled root and foliar inoculations, as well as in commercial field trials.

**Results:**

Screening identified complete resistance in wild *Solanum* accessions and cultivated lines carrying *Tm-1*. Controlled experiments showed that susceptible rootstocks enabled infection of resistant scions following root inoculations, whereas resistant rootstocks limited soil-mediated infection of susceptible scions. In field trials, resistant rootstocks reduced early fruit symptoms. Combining resistant scions with resistant rootstocks (RS/RR) resulted in a 16.43% infection rate, compared to over 92% in susceptible controls.

**Discussion:**

Resistant rootstocks act as a barrier against soil-mediated ToBRFV transmission and reduce economic losses by limiting fruit symptoms. However, they do not protect against direct foliar infection. Thus, combining *Tm-1*-based resistant scions with resistant rootstocks is essential for durable disease management.

## Introduction

Tomato (*Solanum lycopersicum* L.) is a member of the *Solanaceae* family. Worldwide production of tomatoes in 2023 was estimated to be 192 million tons grown over 5.4 million hectares (Fao, 2023). The cultivated tomato, as a self-pollinated crop, lacks genetic diversity and has a germplasm that has been severely depleted by the processes of domestication and breeding of cultivars outside the native zone (Rick and Chetelat, 1995). To alleviate susceptibility to viral diseases, many disease-resistance genes found in wild species of tomato plants were incorporated into commercial tomato lines (Rothan et al., 2019). Wild species of tomato plants were also employed to generate interspecific hybrids for grafting (King et al., 2010). In today’s agriculture, grafting is a common practice used to enhance plant performance under different abiotic and biotic stresses. Self-grafting as a practice to increase plant yield of fruit crops was described in China already in the 5th century and grafting of herbaceous crops such as cucurbits was described in Korea in the 17th century (Lee and Oda, 2003). The first recorded use of interspecific grafting to promote management of soil-borne diseases such as fusarium wilt came from Japan in the 1920’s, where watermelon (*Citrullus lanatus*) was grafted onto squash (*Cucurbita moschata*) (Kubota et al., 2008; Singh and Soltan, 2016; Tateishi, 1927). The first grafting of *Solanaceae* family plants to combat soil borne diseases such as fusarium wilt was of eggplant (*Solanum melongena L.*) grafted on scarlet eggplant (*Solanum integrifolium Poir*) in the 1950s (Oda, 1999). Tomato grafting was introduced commercially in the 1960s (Lee and Oda, 2003). Many studies conducted in recent years demonstrated the positive effect of grafting on enhancement of plant performance such as water-use efficiency, nutrient uptake, as well as fruit yield and quality. In addition, positive effect of grafting was documented on plant response to various abiotic and biotic stresses, the latter include response to bacteria, fungi and viruses (Singh et al., 2017). Regarding the effect on plant response to viruses, reduced virus titer and disease symptoms occurred upon infection of grafted plants with *Cucumovirus cucumbermosaic*, cucumber mosaic virus (CMV), *Orthotospovirus tomatomaculae*, tomato spotted wilt virus (TSWV) or *Potyvirus solani*, potato virus Y (PVY) due to grafting itself or when plants were grafted on resistant rootstocks (Spanò et al., 2020).

Tobamoviruses are major disease-causing pathogens of the *Solanaceae* family, and in particular of tomato cultivation (Ishibashi et al., 2023). Cultivated tomato plants harbor the resistance genes *Tm-1* and the allelic *Tm-2* and *Tm-2^2^*, which were introgressed into the cultivated variety from the wild tomato plants: *Solanum habrochaites* and *S. peruvianum*, respectively (Lanfermeijer et al., 2003, Lanfermeijer et al., 2004) to combat the disease caused by the tomato infecting tobamoviruses, *Tobamovirus tomatomosaicum*, tomato mosaic virus (ToMV) and *tomato mottle mosaic virus* (ToMMV) (Lanfermeijer et al., 2003, Lanfermeijer et al., 2004; Nagai et al., 2019). In recent years, a new tobamovirus emerged, the *Tobamovirus fructirugosum*, tomato brown rugose fruit virus (ToBRFV). The first reports came from Jordan and Israel in 2016 and 2017 (Luria et al., 2017; Salem et al., 2016) and since then a worldwide spread of the virus has occurred (Caruso et al., 2022; Zhang et al., 2022). ToBRFV infects a variety of host plants including tomatoes harboring the *Tm-2^2^* resistance allele (Luria et al., 2017). ToBRFV disease symptoms include a mild to a severe mosaic developed on leaves, narrow leaf tips, as well as curling of the whole leaf and occasional shoestring-like symptoms, which can occur in severely infected plants (Klap et al., 2020). Additionally, yellow/green spots form on infected fruits and in some cases, brown rugose necrotic manifestations developed on fruit surfaces (Luria et al., 2017). Symptoms typically develop within 12 to 18 days post infection, and the disease can result in yield losses of 10-70%, depending on climate conditions and the tomato variety (Oladokun et al., 2019).

Similar to other tobamoviruses (Broadbent, 1976), multiple ways of ToBRFV transmission contribute to viral spread, primarily associated with mechanical means (Ehlers et al., 2023; Panno et al., 2020; Skelton et al., 2023) and preservation in soil (Dombrovsky et al., 2022; Klein et al., 2023; Molad et al., 2024). Soil-mediated ToBRFV transmission could severely affect crops under conditions of monoculture farming. Although the efficiency of soil-mediated tobamovirus transmission is low compared to foliar mechanical infections (Choi et al., 2004; Li et al., 2016), it still poses a threat as a primary source of infectious foci, initiating a secondary foliar mechanical spread throughout the growing season. Soil-mediated ToBRFV transmission occurs *via* root injury when the tobamovirus reaches the upper parts of the plant (Dombrovsky et al., 2022; Klein et al., 2023). The rapid worldwide spread of ToBRFV requires multiple approaches to alleviate the disease. Thus, within the vast genetic biodiversity of tomato wild species, a genetic solution could be found for ToBRFV disease management (Osei et al., 2018; Scott, 2007).

Recent studies have identified wild tomato germplasms showing tolerance or resistance toward ToBRFV (Jaiswal et al., 2024, Jaiswal et al., 2025; Jewehan et al., 2022; Kabas et al., 2022; Rochsar et al., 2025; Topcu et al., 2025; Zinger et al., 2021). Plants tolerant toward the virus were defined as showing no apparent disease symptoms associated with virus titer, whereas resistant plants were defined as restricting virus replication or systemic invasion while manifesting no apparent disease phenotype (Cooper and Jones, 1983; Paudel and Sanfaçon, 2018). In our previous work, we identified and mapped the resistance factor from the wild species *S. pennellii* ‘LA5240’ and the cultivated line ‘Rotem’ (G29884) from the Zamir lab collection to an 89-kb interval on chromosome 2 that contain six genes, including the *tobacco mosaic virus* (TMV) resistance gene *Tm-1* (Rochsar et al., 2025). Recent published results support our findings and validated the role of the *Tm-1* gene as the main resistance conferring gene, while Zinger et al. and Zois et al. described that an additional locus on chromosome 11 is necessary as well (Zinger et al., 2025; Zois et al., 2025).

Considering the contributions of grafted plants to the enhancement of plant performance and the improved efficiency in response to abiotic and biotic stress, the employment of grafting to impede soil-mediated ToBRFV infection could be highly beneficial for commercial crop growers. In the current study, we screened wild and cultivated tomato plants for tolerance/resistance toward ToBRFV and generated *Tm-1*-based rootstocks that could hinder soil-mediated ToBRFV transmission to susceptible cultivated tomato plants.

## Results

### Calibrating a ToBRFV disease-symptom severity scale

**Figure 1:**
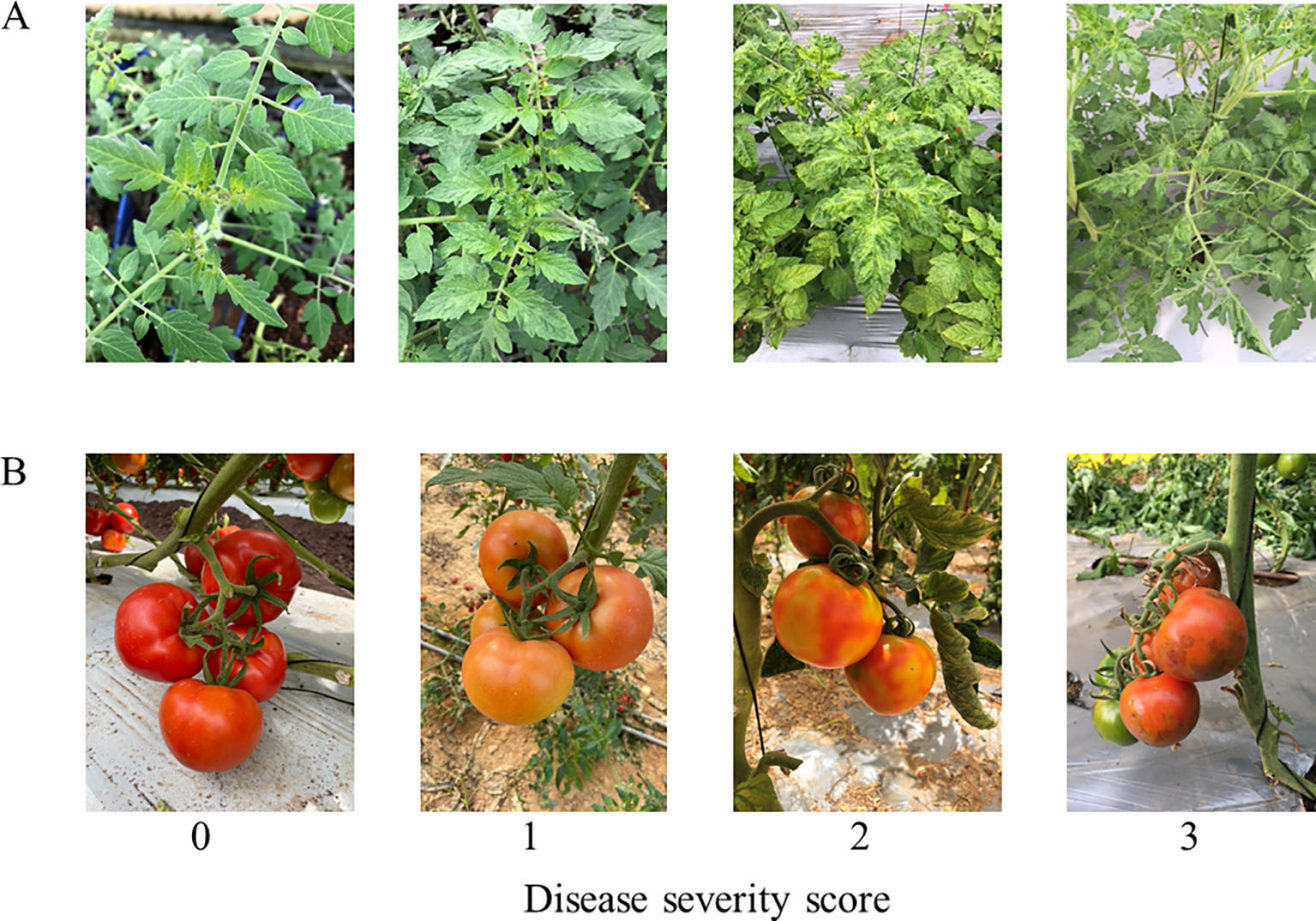
Disease severity scores for tomato plants’ leaves and fruits. **(A)** Disease severity scores for leaf symptoms: 0, asymptomatic. 1, mild mosaic. 2, severe mosaic and yellowing of the leaf veins. 3, narrowing of leaf tips and curling of the whole leaf. **(B)** Disease severity scores for fruit symptoms: 0, asymptomatic. 1, mature fruits not fully ripened. 2, yellow/green spots on fruits accompanied by necrotic lesions on peduncles, pedicels, and the calyces. 3, brown rugose necrotic lesions on fruits.

In order to screen wild and cultivated tomato plants for resistance toward ToBRFV infection, a symptom severity scale of leaves and fruits was established. We examined greenhouse tomato plants naturally infected with ToBRFV in Hatsav village, Israel. We evaluated symptom intensity and severity, manifested on leaves and fruits over a period of 4 months. Disease symptoms included mild to severe mosaic leaves, yellowing leaf veins, narrowing leaf tips, and curled leaves. Fruits showed inhibited ripening, yellow/green blotchy spots, and in some cases, brown rugose necrotic manifestations developed on fruit surfaces. The established severity scale ranged between 0 and 3, from asymptomatic to the most severe symptoms, respectively (**Figure 1**).

### Screening wild tomato accessions for resistance toward ToBRFV inoculations

There is a vast biodiversity within the tomato wild species, therefore, various wild species were employed for introgression of resistance genes into cultivated tomatoes (Rothan et al., 2019).

Analyses of 52 wild species were conducted by scoring plants’ leaf symptoms and calculating each accession’s disease severity index (DSI%). Several accessions from the wild species panel were asymptomatic or showed very low DSI values, which were below 20%. These lines were *S. arcanum* ‘LA1032’, *S. chilense* ‘LA0458’, *S. chilense* ‘LA1917’, *S. chilense* ‘LA1938’, *S. habrochaities* ‘LA0094’, *S. habrochaities* ‘LA1681’, *S. habrochaities* ‘LA2329’, *S. pennellii* ‘LA5240’ (**Table 1**). The samples were also analyzed serologically using enzyme linked immunosorbent assay (ELISA) to determine ToBRFV infection, verifying the plants’ resistance or tolerance toward the virus (**Table 1**). ELISA results showed that most lines were ToBRFV positive. However, three accessions displayed ELISA ratios lower than 3 times the negative reference (3xNR), indicating virus suppression below the detection threshold and were classified as resistant: *S. pennellii* ‘LA0716’, *S. pennellii* ‘LA5240’, and *S. chilense* ‘LA1938’ (**Table 1**). In contrast, accessions such as *S. arcanum* ‘LA1032’ and *S. habrochaites* ‘LA2329’ showed no disease symptoms (DSI = 0) but had detectable viral titers (ELISA ≥ 3xNR), classifying them as tolerant to ToBRFV. Notably, the CAPS marker distinguished between cultivated and wild *Tm-1* alleles but did not differentiate between resistance and nonresistance phenotypes among wild accessions.

**Table 1:** ToBRFV-inoculated wild tomato plants.

Accessions	Source	*DSI(%)	**ELISA OD vs control	*Tm-1* genotype	Accessions	Source	*DSI (%)	**ELISA OD vs control	*Tm-1* genotype
LA 1626	*S. arcanum*	33.3	31.5	*Tm-1/Tm-1*	LA 1625	*S. habrochaities*	55.5	143.3	*Tm-1/Tm-1*
LA 1984	*S. arcanum*	66.7	31.3	*Tm-1/Tm-1*	LA 1681	*S. habrochaities*	0	40.7	*Tm-1/Tm-1*
LA 0378	*S. arcanum*	33.3	11.08	*Tm-1/Tm-1*	LA 1718	*S. habrochaities*	66.7	97.2	*Tm-1/Tm-1*
LA 0441	*S. arcanum*	77.8	36.6	*Tm-1/Tm-1*	LA 1721	*S. habrochaities*	33.3	80.4	*Tm-1/Tm-1*
LA 1032	*S. arcanum*	0	21.1	*Tm-1/Tm-1*	LA 1731	*S. habrochaities*	33.3	11.7	*Tm-1/Tm-1*
LA 1395	*S. arcanum*	22.2	33.7	*Tm-1/Tm-1*	LA 1737	*S. habrochaities*	66.7	57.5	*Tm-1/Tm-1*
LA 2185	*S. arcanum*	66.7	52.9	*Tm-1/Tm-1*	LA 1739	*S. habrochaities*	66.7	65.4	*Tm-1/Tm-1*
LA 2157	*S. arcanum*	N/A	54.2	*Tm-1/Tm-1*	LA 1928	*S. habrochaities*	66.7	69.9	*Tm-1/Tm-1*
LA 2172	*S. arcanum*	N/A	51.8	*Tm-1/Tm-1*	LA 2100	*S. habrochaities*	66.7	217.7	*Tm-1/Tm-1*
LA 0458	*S. chilense*	11.1	15.1	*Tm-1/Tm-1*	LA 2103	*S. habrochaities*	33.3	128.4	*Tm-1/Tm-1*
LA 1917	*S. chilense*	16.7	13.9	*Tm-1/Tm-1*	LA 2106	*S. habrochaities*	66.7	131.9	*N/A*
LA 1938	*S. chilense*	16.7	2.53	*Tm-1/Tm-1*	LA 2155	*S. habrochaities*	50	44.5	*Tm-1/Tm-1*
LA 1317	*S. chmielewskii*	66.7	36.2	*Tm-1/Tm-1*	LA 2196	*S. habrochaities*	66.7	159.3	*Tm-1/Tm-1*
LA 1318	*S. chmielewskii*	66.7	137.8	*Tm-1/Tm-1*	LA 2329	*S. habrochaities*	0	7.03	*Tm-1/Tm-1*
LA 2695	*L.* chmielewskii	N/A	18.2	*Tm-1/Tm-1*	LA 2541	*S. habrochaities*	77.8	180.1	*Tm-1/Tm-1*
LA 2663	*L.* chmielewskii	N/A	25.6	*Tm-1/Tm-1*	LA 2574	*S. habrochaities*	55.5	104.9	*Tm-1/Tm-1*
LA 0746	*L.* cheesmanii	N/A	13.2	*Tm-1/Tm-1*	LA 2357	*S. lycopersicum*	66.7	180.1	*tm-1/tm-1*
LA 0483	*L.* cheesmanii	N/A	9.8	*Tm-1/Tm-1*	LA 1321	*S. neorickii*	66.7	160.1	*Tm-1/Tm-1*
LA 1401	*L.* cheesmanii	N/A	10.5	*Tm-1/Tm-1*	LA 2133	*S. neorickii*	66.7	180.1	*Tm-1/Tm-1*
LA 0094	*S. habrochaities*	11.1	40.5	*Tm-1/Tm-1*	LA 2190	*S. neorickii*	66.7	180.1	*Tm-1/Tm-1*
LA 0407	*S. habrochaities*	44.4	76.02	*Tm-1/Tm-1*	LA 2614	*S. neorickii*	100	57.7	*Tm-1/Tm-1*
LA 1223	*S. habrochaities*	44.4	103.7	*Tm-1/Tm-1*	LA 2732	*S. peruvianum*	44.4	37.6	*Tm-1/Tm-1*
LA 1252	*S. habrochaities*	66.7	43.3	*Tm-1/Tm-1*	LA 4318	*S. peruvianum*	44.4	42.2	*Tm-1/Tm-1*
LA 1298	*S. habrochaities*	33.3	121.5	*Tm-1/Tm-1*	LA 4325	*S. peruvianum*	66.7	50.6	*Tm-1/Tm-1*
LA 1352	*S. habrochaities*	55.5	30.3	*Tm-1/Tm-1*	LA 5240	*S. pennellii*	0	1.23	*Tm-1/Tm-1*
LA 1362	*S. habrochaities*	55.5	122.1	*Tm-1/Tm-1*	LA 716	*S. pennellii*	N/A	2.5	*Tm-1/Tm-1*

*DSI, disease severity index, values below 20% were considered as indicative of tolerance. **ELISA OD vs control, ratios lower than 3xNR (negative reference) were considered ToBRFV negative whereas values higher than 3xNR were considered ToBRFV positive.

### Screening cultivated tomato lines for resistance toward ToBRFV inoculations

Screening 4**5** cultivated and commercially available lines for resistance toward ToBRFV was conducted by growing the plants in a commercial greenhouse in Mivtahim village, Israel. Following ToBRFV foliar inoculations, phenotypes for ToBRFV disease symptoms of leaves and fruits were determined over a 4-month growth period. Nine cultivated lines were asymptomatic (DSI = 0 for both leaves and fruits) and ELISA-negative (OD < 3xNR), indicating complete resistance to ToBRFV (**Table 2**). These lines were: ‘Rotem’ (G29884), ‘Davis_1’, ‘Davis_2’, ‘GPT214320’, the F_3_ line ‘33’, and the hybrids ‘NBK_20227’, ‘NBR_21784’, ‘NBR_22’, and ‘NBM_80’, all of which harbor the *Tm-1* allele in either a heterozygous or homozygous mode of inheritance.

**Table 2:** ToBRFV-inoculated cultivated tomato plants.

Cultivar	*DSI leaf (%)	*DSI fruit (%)	**ELISA OD vs control	*Tm-1* genotype	Cultivar	*DSI leaf (%)	*DSI fruit (%)	**ELISA OD vs control	*Tm-1* genotype
33	0	0	1.3	*Tm-1/Tm-1*	M-82	46.6	0	16.3	*tm-1/tm-1*
4755	66.6	33.3	18.02	*tm-1/tm-1*	Magallanes	44.4	22.2	N/A	*tm-1/tm-1*
Almina	33.3	0	N/A	*tm-1/tm-1*	Miamour	66.6	N/A	N/A	*tm-1/tm-1*
Antamay	51.8	25.9	N/A	*tm-1/tm-1*	NBM_80	0	0	2.99	*Tm-1/tm-1*
Ateneo	66.6	100	N/A	*tm-1/tm-1*	NBK_20227	0	3.33	1.9	*Tm-1/tm-1*
Attiya	33.3	0	N/A	*tm-1/tm-1*	NBR_21784	0	5.5	1.95	*Tm-1/tm-1*
Bosco	80	N\a	N/A	*tm-1/tm-1*	NBR_22	0	14.2	1.09	*Tm-1/tm-1*
By Elsa	46.1	30.7	N/A	*tm-1/tm-1*	Odelia	66.6	33.3	72.9	*tm-1/tm-1*
DAVIS_1	0	0	0.8	*Tm-1/Tm-1*	Ramyle	73.3	33.3	N/A	*tm-1/tm-1*
DAVIS_2	0	0	0.7	*Tm-1/Tm-1*	Razimo	66.6	0	N/A	*tm-1/tm-1*
Eshkol	50	0	N/A	*tm-1/tm-1*	Redgard	83.3	0	24.5	*tm-1/tm-1*
FreshTom	66.6	N/A	N/A	*tm-1/tm-1*	Santa west	40	N/A	N/A	*tm-1/tm-1*
Rotem (G29884)	0	0	1.1	*Tm-1/Tm-1*	Shani	66.6	0	N/A	*tm-1/tm-1*
GPT200700	66.6	33.3	19.2	*tm-1/tm-1*	Shiren	100	0	22	*tm-1/tm-1*
GPT202680	33.3	33.3	64.3	*tm-1/tm-1*	Simon	100	33.3	18.6	*tm-1/tm-1*
GPT214320	0	0	2.1	*Tm-1/Tm-1*	Top	53.3	26.6	32.9	*tm-1/tm-1*
Glory TY	66.6	N/A	N/A	*tm-1/tm-1*	Torry	44.4	0	44.9	*tm-1/tm-1*
Heinz 1662	55.5	30	N/A	*tm-1/tm-1*	Ventero	66.6	33.3	N/A	*tm-1/tm-1*
Ikram	100	33.3	16	*tm-1/tm-1*	Vernal	100	0	N/A	*tm-1/tm-1*
K-115	100	66.6	23.3	*tm-1/tm-1*	Whitney	100	0	26.3	*tm-1/tm-1*
Lea	66.6	100	50.2	*tm-1/tm-1*	Yeniceri	100	N/A	N/A	*tm-1/tm-1*
Lobello	66.6	0	23.8	*tm-1/tm-1*	YO-1	100	N/A	N/A	*tm-1/tm-1*
					Zohara	100	0	13.2	*tm-1/tm-1*

*DSI, disease severity index, values below 20% were considered as indicative of tolerance. **ELISA OD vs control, ratios lower than 3xNR (negative reference) were considered ToBRFV negative whereas values higher than 3xNR were considered ToBRFV positive.

### Comparative analysis of *Tm-1* alleles revealed variations in the virus replicase binding domain

From the resistant accessions identified in our screening, we selected the wild species *S. pennellii* ‘LA5240’ and the cultivated line ‘Rotem’ (G29884) for further investigation. LA5240 was chosen for its high cross-compatibility with cultivated backgrounds, and both LA5240 and ‘Rotem’ were previously used in F1 and F2 populations for validation of the *Tm-1* locus (Rochsar et al., 2025). In addition, the resistant wild *S. pennellii* accession LA716 was included for comparative sequence analysis. We compared the *Tm-1* amino acid sequences of *S. pennellii* ‘LA5240’ (*Tm-1^LA5240^*), LA716 (*Tm-1^LA716^*), and Rotem (G29884) (*Tm-1^Rotem^*) and found that the resistant alleles shared high similarity with each other: *Tm-1^LA5240^* and *Tm-1^LA716^* were 99.86% identical, and *Tm-1^Rotem^* showed 98.85% identity with LA5240 and 98.94% with LA716. All three resistant alleles shared 89–90% identity with the susceptible *tm-1* allele (**Supplementary Figure 1**).

Interestingly, the binding site to the tobamovirus ToMV replicase protein, located between amino acid positions 79 and 112 on the NN-domain (Ishibashi et al., 2014), was identical between *Tm-1^LA5240^* and *Tm-1^LA716^* (100%), and showed 94.12% identity with *Tm-1^Rotem^*. In contrast, identity to the susceptible *tm-1* allele was lower: 76.47% for *Tm-1^LA5240^* and *Tm-1^LA716^*, and 70.59% for *Tm-1^Rotem^* (**Supplementary Figure 1**).

In addition, there is a sixty–amino acid deletion between positions 420 and 480 in the *Tm-1^LA5240^* allele. Comparative analysis of F_2_ progenies of the two allelic variants derived from ‘LA5240’ and ‘Rotem (G29884)’ revealed no significant difference in their capacity to confer resistance (Rochsar et al., 2025). In the current study, hybrid lines generated from *S*. *pennellii* ‘LA5240’ and Rotem (G29884) similarly displayed resistance toward ToBRFV inoculations (**Table 2** and see below).

### Assessing soil-mediated ToBRFV infection of *Tm-2^2^*-resistant tomato plants, planted in soil of a previous growth cycle of an infected crop

**Figure 2:**
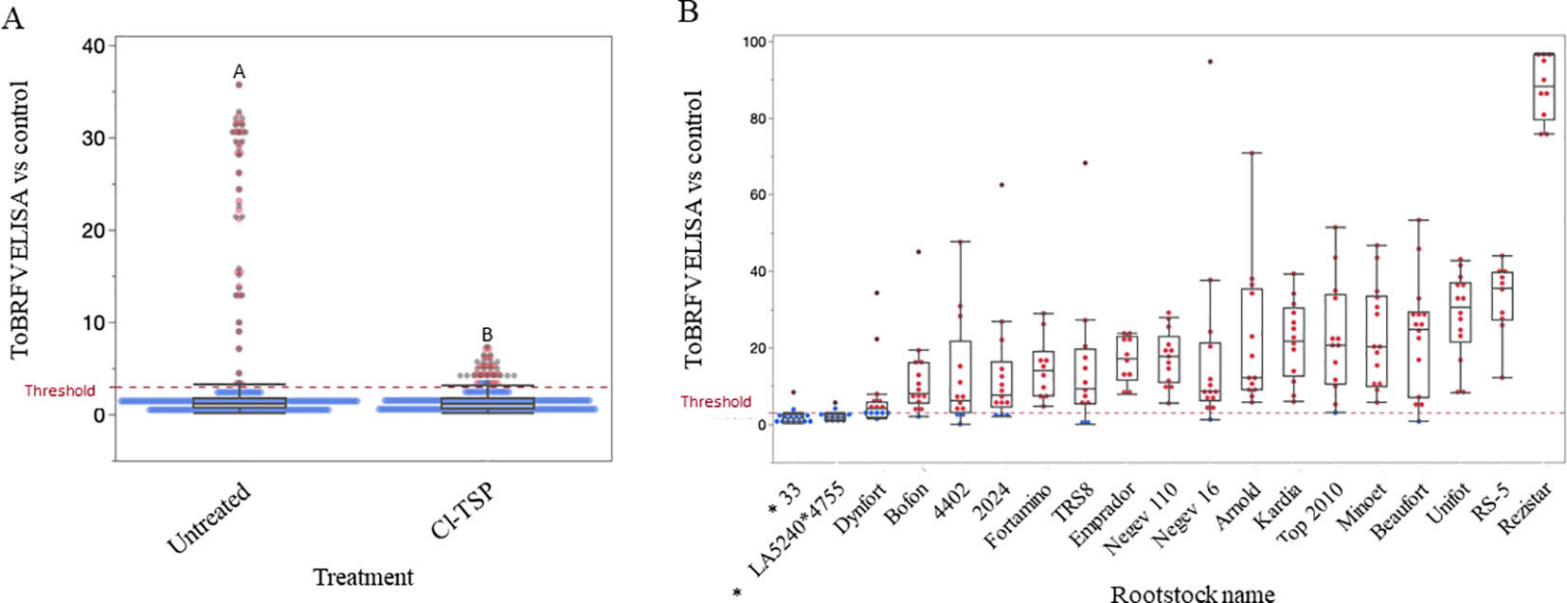
Impact of soil treatment and rootstock origin on ToBRFV infection. **(A)** ToBRFV ELISA results of susceptible tomato plants (cv. Ikram), at 21 days post regular planting, conducted in a greenhouse experiment in soil of a previous growth cycle of infected crops. Planting either was in untreated soil-pits or treated with 3% chlorinated tri-sodium phosphate (Cl-TSP). Different letters indicate statistical differences between results, using t-Test (P<0.05). **(B)** ToBRFV ELISA results of foliar-inoculated rootstocks comparing the commercially available rootstocks with those generated from *S. pennellii* ‘LA5240’ and Rotem crosses (**Figure 3**). The red dotted lines in the graphs indicate the threshold of 3xNR (negative reference). Red dots indicate positive ELISA results showing ratios higher than 3xNR, and blue dots indicate negative ELISA results showing ratios lower than 3xNR. A black line indicates the mean of each treatment. A star (*) next to a rootstock name indicates statistically significant differences between the tested lines using Tukey HSD test (P<0.05).

We have previously shown that soil of ToBRFV-infected crops contained infectious virus, which was demonstrated by symptom development in root-truncated newly planted *Tm-2^2^*-resistant tomato plants (Molad et al., 2024). We have also shown that soil disinfection with 3% chlorinated tri-sodium phosphate (Cl-TSP) reduced soil infectivity (Dombrovsky et al., 2022; Molad et al., 2024). We conducted a large-scale experiment in which *Tm-2²*-resistant tomato plants were planted in soil from a previous cycle of ToBRFV-infected plants to assess soil-mediated infection under realistic greenhouse conditions. A total of 384 plants were divided into two groups; one was planted in the contaminated soil and the second was planted in Cl-TSP-treated contaminated soil. The plants were grown for 21 days and then subjected to ELISA test. The results showed that the newly planted tomato plants were ToBRFV-infected with a mean ELISA value of 4.15xNR (negative reference), whereas the plants planted in the Cl-TSP-treated soil showed significantly lower ToBRFV infection with the mean value of 1.5xNR (**Figure 2**). However, the Cl-TSP treatment did not totally prevent infection of the newly planted tomato plants with 12.5% of the plants showing ToBRFV infection (**Figure 2**).

### Analyses of rootstock resistance toward ToBRFV inoculations

Two hybrid crosses were generated, using the wild resistant line *S. pennellii* ‘LA5240’ and the cultivated line Rotem: LA5240*4755 and ‘33’, respectively (see below). We tested the two hybrid crosses along with a panel of 17 of the leading commercial rootstocks available in the market for susceptibility to ToBRFV infection *via* foliar inoculations, four weeks following sowing. ELISA test conducted at 30 days post inoculations (dpi) revealed that the two rootstocks ‘LA5240*4755’ and ‘33’, were resistant to ToBRFV, showing ELISA values significantly different from all the tested commercial rootstocks using the Tukey HSD test (**Figure 2B**). For the rest of our experiments, we used the commercial Beaufort, RS-5, Kardia and Rezistar as susceptible rootstocks.

### Generating resistant scions and rootstocks

**Figure 3:**
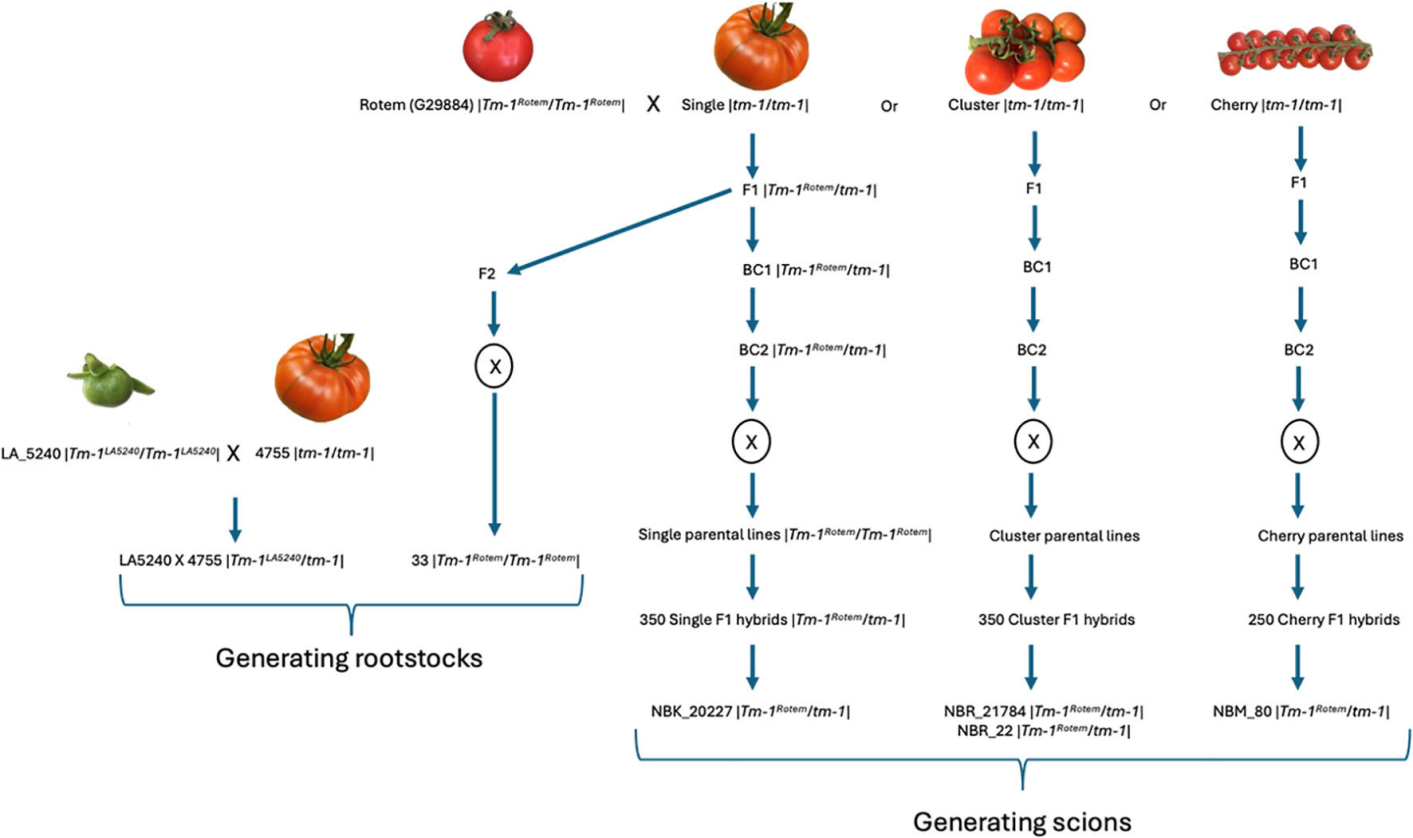
Schematic overview of the breeding strategy used to develop *Tm-1*–based resistant rootstocks and scions. For developing resistant rootstocks, the wild species *S. pennellii* ‘LA5240’ was used as the donor of the resistant *Tm-1^LA5240^* allele and was crossed with the susceptible line ‘4755’ to create heterozygote F1 hybrids. The cultivated line ‘Rotem’ (G29884) served as the donor of the resistant *Tm-1^Rotem^* allele and was crossed with susceptible lines from the single-pick, cluster, and cherry tomato fresh market segments, each carrying the susceptible *tm-1* allele. We selected the F_3_ line ‘33’ as a rootstock as well. To generate scions, two consecutive backcrosses (BC_1_, BC_2_), followed by self-pollination (X), were performed to obtain parental lines homozygous for *Tm-1^G^.* These parental lines were then crossed with multiple susceptible lines within each segment to produce F_1_ hybrids heterozygous for *Tm-1*. *Tm-1* genotype is presented next to each breeding stage and the source of each allele is indicated as well, where *Tm-1* originated from *S. pennellii* ‘LA5240’ was marked as *Tm-1^LA5240^*, the one originated from ‘Rotem’ (G29884) was marked as *Tm-1^Rotem^* and the susceptible allele was marked as *tm-1*.

To evaluate the effect of grafting on ToBRFV infection either *via* root or foliar inoculations, we generated a series of resistant scions and rootstocks. We used the wild species *S. pennellii* ‘LA5240’ and the cultivated line Rotem (G29884) as donors of the *Tm-1*-based resistance trait. To develop resistant scions, the cultivated line ‘Rotem’ (accession G29884) was crossed with several susceptible cultivated lines representing different tomato market segments: single-pick, cluster, and cherry tomatoes from Prof. Zamir’s breeding program (**Figure 3**). The resulting F_1_ hybrids were backcrossed twice (BC_2_) to the susceptible parental backgrounds. Plants carrying *Tm-1* were identified using a single nucleotide polymorphism (SNP) marker (Rochsar et al., 2025), and self-pollination of the selected BC_2_ plants was carried out to generate homozygous *Tm-1* parental lines. These homozygous resistant parental lines were then crossed with multiple susceptible parental lines across the different tomato segments to produce F_1_ hybrids heterozygous for *Tm-1*: 350 single-pick, 350 cluster, and 250 cherry tomato hybrids. From these tomato hybrids, we selected four lines that displayed resistance to ToBRFV and had desirable agronomic traits such as fruit shape, size, and yield. The selected hybrids included ‘NBK_20227’ (of the single-pick segment), ‘NBR_21784’ and ‘NBR_22’ (of the cluster segment), ‘NBM_80’ (of the cherry segment), and the homozygote F_3_ line ‘33’ (**Figure 3**). These selected lines were added to the panel of cultivated tomato lines (**Table 2**) and served as resistant scions in our grafting experiments. To generate resistant interspecific rootstock, the wild species *S. pennellii* ‘LA5240’ was crossed with the susceptible line ‘4755’ to produce the LA5240×4755 hybrid. Line ‘33’ was also evaluated directly as a rootstock.

### Root inoculations of susceptible rootstocks overcame ToBRFV resistance of scions

**Figure 4:**
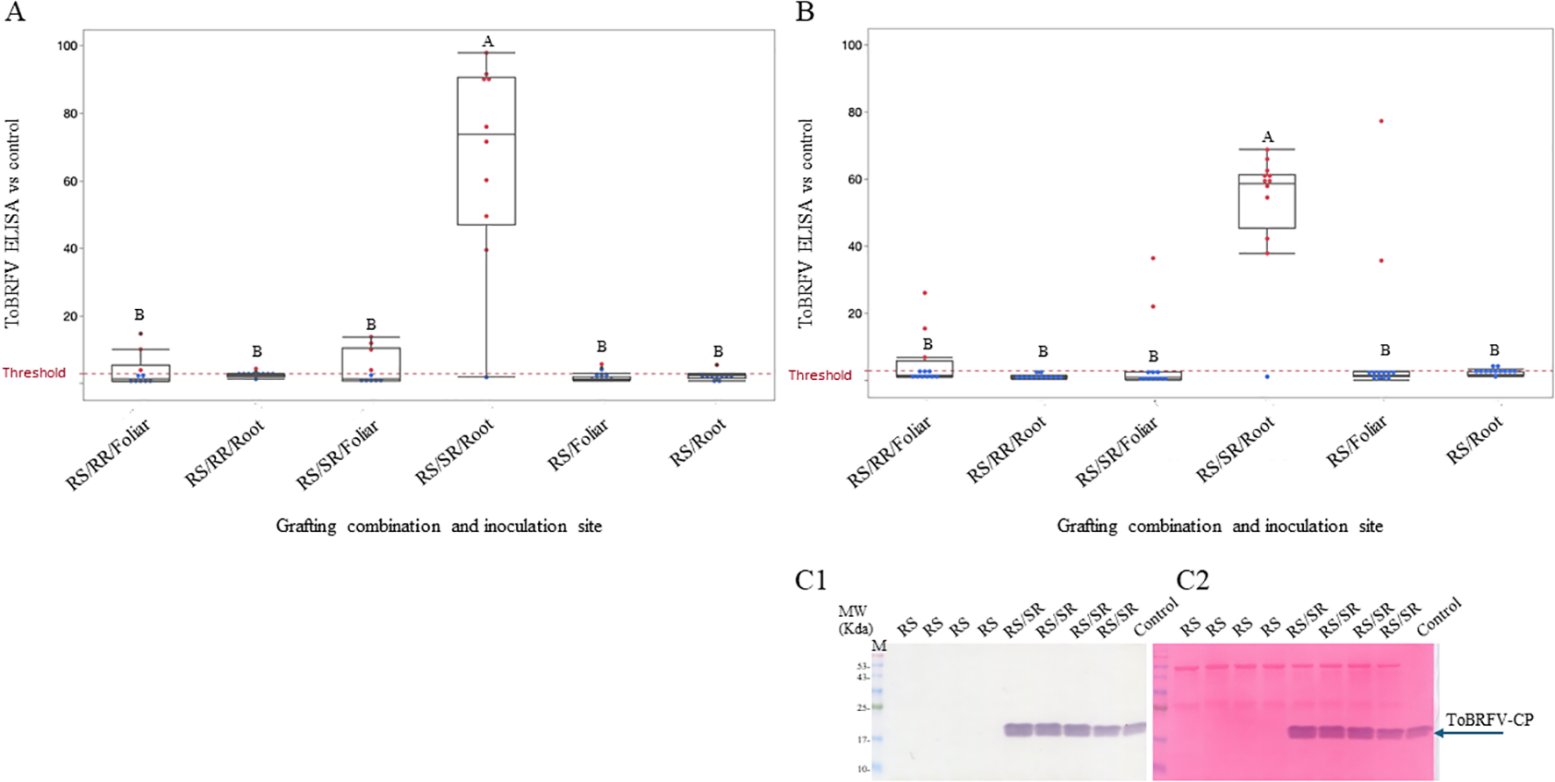
ToBRFV root inoculations of a susceptible rootstock overcame scion resistance toward ToBRFV. **(A)** ToBRFV ELISA results of foliar- or root-inoculated resistant scion ‘NBK_20227’, un-grafted (RS), grafted on either the resistant rootstock ‘LA5240*4755’ (RS/RR) or the susceptible rootstock ‘RS-5’ (RS/SR), grown during the spring season. **(B)** ToBRFV ELISA results of foliar- or root-inoculated resistant scion ‘NBR_21784’, un-grafted (RS), grafted on either the resistant rootstock ‘LA5240*4755’ (RS/RR) or the susceptible rootstock ‘Rezistar’ (RS/SR), grown during the fall season. The red dotted lines indicate the threshold of 3xNR (negative reference). Red dots indicate positive ELISA results with ratios higher than 3xNR, and blue dots indicate negative ELISA results with ratios lower than 3xNR. A black horizontal line in each box indicates the mean of each treatment. Different letters indicate significant differences among treatments based on a General Linear Model followed by Tukey’s HSD test (p < 0.05). RS, resistant scion; RR, resistant rootstock; SR, susceptible rootstock. C1. Quantification of ToBRFV by a western blot showing sampled un-grafted ‘NBR_21784’ (lanes 1-4) and ‘NBR_21784’ grafted on ‘Rezistar’ following root inoculations (lanes 5-8). A positive control of a ToBRFV-infected tomato leaf is marked ‘control’. C2. Ponceau-s staining showing equal loading. M, molecular size marker; CP, coat protein.

In an experiment conducted during the spring of 2022, the cultivated resistant tomato hybrid ‘NBK_20227’ showed resistance toward ToBRFV foliar inoculations (**Figures 4A** RS) with a mean ELISA ratio of 1.7xNR. Similarly, upon root inoculations, the mean ELISA result was 2.27xNR. The resistant scion (RS) ‘NBK_20227’ grafted on the susceptible rootstock (SR) ‘RS-5’ did not reduce plant infection by ToBRFV upon root inoculations (**Figure 4A**, RS/SR). The mean ELISA result of the infected scion of the root-inoculated RS/SR grafted plant was 66.7xNR. Foliar inoculations of these RS/SR-grafted plants limited ToBRFV infection showing a mean ELISA result of 4.5xNR, not significantly different from foliar inoculations of RS/RR-grafted plants with 3.5xNR (**Figure 4A**). Statistical analysis using a General Linear Model (multifactor ANOVA), including grafting combinations, inoculation methods, and their interactions, revealed no significant differences between foliar and root inoculations in RS/RR-grafted plants. *Post hoc* comparisons of LSMeans using Tukey’s HSD test (p < 0.05) supported these observations. Similar results were observed in an experiment conducted during the fall of 2024 in which we employed ‘NBR_21784’ as the resistant scion (RS), and ‘Rezistar’ as a susceptible rootstock (SR) (**Figure 4B**). In both experiments, the resistant rootstock ‘LA5240*4755’ limited ToBRFV infection of the resistant scion upon root inoculations (**Figures 4A, B**, RS/RR). A western blot analysis demonstrates ToBRFV infection of sampled plants with a resistant scion grafted on a susceptible rootstock, subjected to ToBRFV root inoculations (**Figures 4C1, C2**).

### Resistant rootstocks hindered ToBRFV infection of susceptible scions upon root inoculations

**Figure 5:**
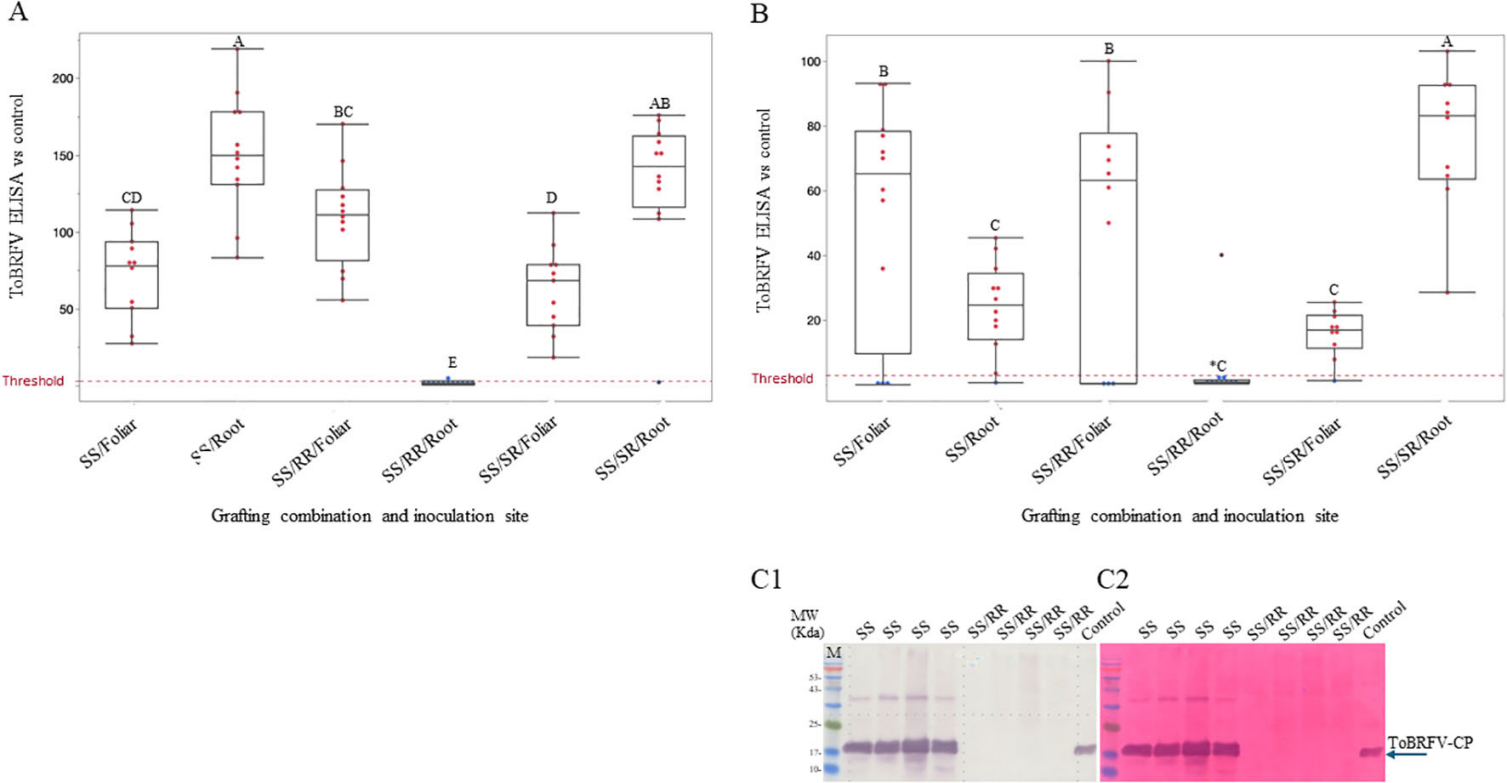
Resistant rootstocks hindered ToBRFV infection of susceptible scions upon root inoculations. **(A)** ToBRFV ELISA results of foliar- or root-inoculations of susceptible ‘Odelia’ un-grafted (SS), grafted on a resistant line ‘33’ (SS/RR) or self-grafted (SS\SR). The experiment was conducted under stringent conditions of high ToBRFV concentrations as well as root truncations. **(B)** ToBRFV ELISA results of the susceptible scion ‘Shiren’, un-grafted (SS), grafted on either a resistant rootstock ‘LA5240*4755’ (SS/RR), or a susceptible rootstock ‘Rezistar’ (SS/SR). A dotted red line indicates the threshold of 3xNR (negative reference). Red dots indicate positive ELISA results showing ratios higher than 3xNR, and blue dots indicate negative ELISA results showing ratios lower than 3xNR. A black horizontal line in each box indicates the mean absorbance of each treatment group. Different letters indicate significant differences among treatments based on a General Linear Model followed by Tukey’s HSD test (p < 0.05). A star (*) next to connecting letter indicate statistical difference between this treatment and the treatments that carry the same letter using t-Test (P<0.05). SS, susceptible scion; RR, resistant rootstock; SR, susceptible rootstock. **(C1)**. Quantification of ToBRFV by a western blot showing un-grafted ‘Shiren’ plants (lanes 1-4) and ‘Shiren’ plants grafted on ‘LA5240*4755’ following root inoculations (lanes 5-8). A positive control of a ToBRFV-infected tomato leaf is marked as ‘control’. **(C2)**. Ponceau-s staining showing equal loading. M, molecular size marker; CP, coat protein.

In order to generate grafted plants, line ‘33’, resistant to ToBRFV inoculations, was employed as a rootstock (resistant rootstock, RR) to test its effect on ToBRFV infection of the susceptible scion (SS) ‘Odelia’ upon root or foliar inoculation (**Figure 5A**). Truncated roots of the cultivated line ‘33’ were dipped in ToBRFV sap to increase stringency of the inoculation test. The results showed that line ‘33’ hindered soil-mediated ToBRFV infection of the susceptible ‘Odelia’ scion, showing a mean ELISA value of 1.52xNR. The un-grafted ‘Odelia’ plants showed susceptibility to ToBRFV infection upon foliar or root inoculations, with the mean ELISA values of 72.9xNR and 150.49xNR, respectively. However, the resistant ‘33’ rootstock did not prevent ToBRFV infection of the ‘Odelia’ scion upon foliar inoculations showing ELISA values of 109.5xNR (**Figure 5A**). Self-grafting of ‘Odelia’ did not affect the plant response to ToBRFV inoculations, either by foliar or root inoculations, showing mean ELISA values of 62.7xNR and 132.5xNR, respectively. Statistical analysis was performed using a General Linear Model (multifactor ANOVA), including grafting combination, inoculation method, and their interaction. *Post hoc* comparisons of LSMeans using Tukey’s HSD test (p < 0.05) revealed that ToBRFV infection upon root inoculations of susceptible ‘Odelia’ plants was significantly higher than upon foliar inoculations, highlighting the stringency of the root inoculation test.

In an experiment conducted during spring of 2022, we tested the effectivity of the resistant rootstock ‘LA5240*4755’ to hinder soil-mediated ToBRFV infection of susceptible scions, as was observed for the resistant line ‘33’. As a positive control, the susceptible ‘Shiren’ variety of cultivated tomatoes served as scions grafted on the susceptible rootstocks ‘Rezistar’ (**Figure 5B**, SS/SR). Upon root inoculations, ToBRFV infected the grafted plants of the positive control showing a mean ELISA result of 76.3xNR. Under similar conditions of root inoculations, when the susceptible ‘Shiren’ was grafted on the resistant rootstock ‘LA5240*4755’, the rootstock limited ToBRFV infection of the susceptible scion ‘Shiren’, showing mean ELISA result of 0.87xNR (excluding one of the ten plants, **Figure 5B**, SS/RR). However, under similar conditions of using a high inoculum of ToBRFV, the resistant rootstock ‘LA5240*4755’ did not overcome ToBRFV foliar inoculations of the susceptible scion ‘Shiren’, and ToBRFV infected the plants showing a mean ELISA result of 51.1xNR. A western blot analysis showed the effect of the resistant rootstock ‘LA5240*4755’ on limiting ToBRFV infection of the susceptible scion ‘Shiren’ upon root inoculations (**Figures 5C1, C2**).

**Figure 6:**
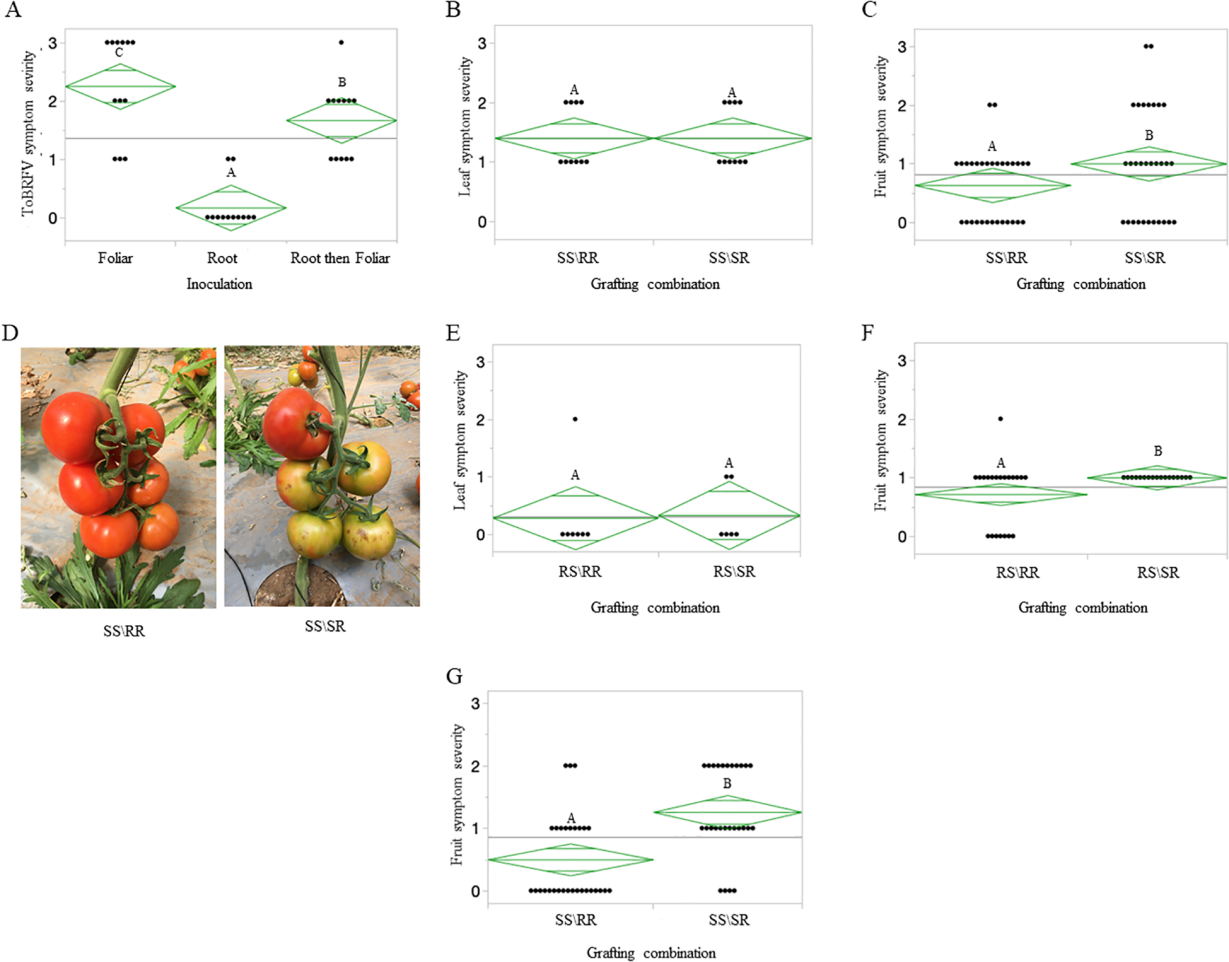
Resistant rootstocks mitigate ToBRFV symptoms under combined root and foliar inoculations and in early fruits in a field trial **(A)** ToBRFV disease-symptom severity of the susceptible scion ‘Shiren’ grafted on the resistant rootstock ‘LA5240*4755’ (SS/RR), subjected to foliar inoculations, root inoculations, or foliar inoculations preceded by root inoculations **(B-G)**. Field trials. **(B)** Disease-symptom severity scores of leaves of susceptible ‘Odelia’ grafted on either the susceptible rootstock ‘Beaufort’ (SS/SR) or the resistant rootstock ‘LA5240*4755’ (SS/RR). **(C)** Disease-symptom severity scores of fruits of the first three clusters of susceptible ‘Odelia’ grafted either on the susceptible rootstock ‘Beaufort’ (SS/SR) or the resistant rootstock ‘LA5240*4755’ (SS/RR). **(D)** Representative pictures of the first cluster of ‘Odelia’ grafted on ‘Beaufort’ (SS\SR), and ‘Odelia’ grafted on ‘LA5240*4755’ (SS\RR). **(E)** Disease-symptom severity scores of leaves of the resistant ‘NBR_22’ grafted on either the susceptible rootstock ‘Beaufort’ (RS/SR) or the resistant rootstock ‘LA5240*4755’ (RS/RR). **(F)** Disease-symptom severity scores of fruits of the first three clusters of the resistant ‘NBR_22’ grafted on the susceptible rootstock ‘Beaufort’ (RS/SR) or the resistant rootstock ‘LA5240*4755’ (RS/RR). **(G)** Disease-symptom severity scores of fruits of susceptible scion ‘Odelia’ grafted either on the susceptible rootstock ‘Kardia’ (SS/SR) or the resistant rootstock ‘LA5240*4755’ (SS/RR). A green horizontal line crossing the diamond-shape data indicates mean of each result, and different letters indicate statistically significant differences between the results using student t-Test (P<0.05) for A and t-Test (P<0.05) for **(B, C, E-G)** SS, susceptible scion; SR, susceptible rootstock; RS, resistant scion; RR, resistant rootstock.

In an experiment conducted during fall of 2024, we have shown that indeed, the resistant rootstock ‘LA5240*4755’ limited ToBRFV infection of the susceptible scion ‘Shiren’ upon root inoculations, showing a mean ELISA result of 1.1xNR and the mean disease-symptom severity score was 0.16 with DSI values of 5.5% (**Figure 6A**). In this experiment, to initiate a defense response by the resistant rootstocks, we first root-inoculated the rootstocks with ToBRFV before subjecting the susceptible ‘Shiren’ scions to foliar inoculations. A mean ToBRFV ELISA value observed upon foliar inoculations of the combined ToBRFV-inoculated grafted plants was 18.5xNR compared with the foliar-only inoculated grafted plants, showing a mean ELISA value of 25.7xNR. The effect of combined ToBRFV inoculations on reducing viral levels, tested by ELISA, was not statistically significant. However, the combined ToBRFV inoculations significantly reduced the disease-symptom severity observed on the grafted plants (**Figure 6A**). A mean disease-symptom severity score of 1.6, with DSI values of 55.5%, was observed in the grafted plants subjected to combined ToBRFV inoculations, whereas grafted plants subjected to foliar inoculations only showed a mean disease-symptom severity score of 2.25 with DSI values of 75%.

### In a field trial, resistant rootstocks reduced ToBRFV associated fruit symptom severity of susceptible scions while susceptible rootstocks increased the fruit symptom severity of resistant scions

In a field trial of grafted plants grown to the fruiting stage, conducted during spring of 2021, plants that were exposed to ToBRFV infection *via* commonly practiced plant manipulations as well as soil-mediated infections were tested for leaf- and fruit-symptom manifestations (**Figures 6B–F**). Disease symptom severity manifestations on the grafted plant’s leaves were not affected by the rootstock used, and the values were similarly high in the susceptible ‘Odelia’ scions grafted on either the resistant rootstock ‘LA5240*4755’ (SS/RR) or the susceptible rootstock ‘Beaufort’ (SS/SR), showing a similar mean value of 1.4 (DSI = 46.6%) (**Figure 6B**). However, the resistant rootstock ‘LA5240*4755’ did confer a significant reduction in disease symptom severity manifested on fruits of the first three clusters of the susceptible ‘Odelia’ scions (SS/RR), showing a mean value of 0.53 (DSI = 21.1%) (**Figures 6C, D**). Disease symptom severity observed on fruits of the ‘Odelia’ scions grafted on the susceptible rootstock ‘Beaufort’ (SS/SR) showed a mean value of 1.0 (DSI = 33.3%) (**Figure 6C**). Similarly, disease symptom severity manifestations on leaves of the resistant scion ‘NBR_22’ were low but independent of the rootstock used, either the resistant rootstock ‘LA5240*4755’ (RS/RR), or the susceptible rootstock ‘Beaufort’ (RS/SR), showing the mean values of 0.28 (DSI = 9.5%) and 0.33 (DSI = 11.1%), respectively (**Figure 6E**). Importantly, disease symptom severity manifested on leaves of the susceptible scions was significantly higher (p<0.05, paired t-Test), from that of the resistant scions (**Figures 6B, E**). However, fruits of the first three clusters of the grafted RS/SR plants showed significantly higher (p<0.05, paired t-Test) disease symptom severity with a mean value of 1.0 (DSI = 33.3%) compared with a mean value of 0.71 (DSI = 23.8%) observed on fruits of the RS/RR plants (**Figure 6F**). In addition, fruit symptom severity scores of SS/RR plants and RS/RR plants were similar (**Figures 6C, F**) (p<0.3141, paired t-Test), indicating a role for the root system in fruit symptom development.

The experiment was repeated in the following growing season using the susceptible scion ‘Odelia’ grafted onto either the susceptible rootstock ‘Kardia’ or the resistant rootstock ‘LA5240×4755’, and fruit symptom development was monitored throughout the growing season. When ‘Odelia’ was grafted onto the susceptible rootstock, the mean fruit symptom score was 1.25 (DSI = 41.9%). In contrast, grafting onto the resistant rootstock resulted in a significantly lower mean symptom score of 0.5 (DSI = 16.6%) (**Figure 6G**).

### Resistant rootstocks reduced ToBRFV infection of susceptible scions in field observations of grafted plants exposed to routine plant manipulations

We have conducted field observations during the years 2020–2023 in several locations around Israel during the fall and spring growing seasons. Grafting combinations that were tested included RS/RR, SS/SR, and SS/RR. The resistant scions were ‘NBK_20227’, ‘NBR_21784’, ‘NBR_22’, and ‘NBM_80’, and the resistant rootstock was ‘LA5240*4755’. The susceptible scions were ‘Shiren’, ‘Ikram’, or ‘Tori’ and the susceptible rootstocks were ‘Beaufort’ or ‘RS-5’ (**Table 3**). Each grafting combination in the trials (70–160 plants for each grafting combination) was randomly sampled for ELISA testing during the growing season (N = 10–80 samples). Across all seven field observations, the percentage of ToBRFV-positive plants as determined by ELISA was consistently lowest in plants grafted with a resistant scion on a resistant rootstock (RS/RR) compared with plants grafted with a susceptible scion on a susceptible rootstock (SS/SR) or ungrafted susceptible plants (SS/X) (**Table 3**). In three field observations conducted in Yated (Y), Amioz (A) and Kadesh Barnea (KB), an additional treatment of susceptible scions grafted onto resistant rootstocks (SS/RR) was evaluated. In Yated and Amioz, SS/RR plants exhibited a statistically significant difference in ELISA-positive plants that were 73.33% and 70% respectively, compared with the SS/SR combination that were 100% ToBRFV positive in both fields. In contrast, no significant difference between SS/RR and SS/SR plants was observed in Kadesh Barnea (KB), where approximately 80% of plants in both treatments tested positive for ToBRFV (**Table 3**). Overall, only 16.43% of RS/RR plants (N = 213) were ELISA positive, whereas 94.67% (N = 150) of SS/SR plants and 92.54% (N = 67) of SS/X plants tested positive (**Table 3**). Susceptible scions grafted onto resistant rootstocks (SS/RR) showed an intermediate infection level, with 73.33% of plants (N = 60) testing positive overall, however, this treatment differed significantly from the SS/SR group in only two of the three field observations.

**Table 3:** Field observations of grafted plants.

Grafting combination	Segment	Scion	Rootstock	Location	N tested	ToBRFV ELISA positive %	Growing season	*Connecting letters
RS/RR	cluster	NBR_21874	LA5240*4755	RN	80	38%	fall 20	A
SS/SR	cluster	Ikram	Beufort	RN	80	100%	fall 20	B
SS/X	cluster	Ikram	ungrafted	M	45	90%	fall 20	A
RS/RR	cluster	NBR_21874	LA5240*4755	M	45	9%	fall 20	B
RS/RR	cluster	NBR_22	LA5240*4755	A	10	10%	fall 20	A
SS/RR	cluster	Ikram	LA5240*4755	A	20	70%	fall 20	B
SS/SR	cluster	Ikram	Beufort	A	20	100%	fall 20	C
SS/RR	cluster	Ikram	LA5240*4755	Y	30	73.33%	spring 21	B
SS/SR	cluster	Ikram	Beufort	Y	10	100%	spring 21	A
RS/RR	cluster	NBR_21874	LA5240*4755	Y	20	0%	spring 21	C
SS/SR	single	Torry	Beufort	P	10	90%	fall 22	A
RS/RR	single	NBK_20227	LA5240*4755	P	10	0%	fall 22	B
SS/RR	cherry	Shiran	LA5240*4755	KB	10	80%	fall 22	A
RS/RR	cherry	NBM_80	LA5240*4755	KB	26	0%	fall 22	B
SS/SR	cherry	Shiran	RS-5	KB	20	80%	fall 22	A
SS/X	cluster	Ikram	ungrafted	BE	22	100%	fall 23	A
RS/RR	cluster	NBR_21874	LA5240*4755	BE	22	0%	fall 23	B

*paired samples subjected to Chi-square/Pearson test for each field observation. RS/RR (resistance scion grafted on resistant rootstock). SS/RR (susceptible scion grafted on resistant rootstock). SS/SR (susceptible scion grafted on susceptible rootstock). SS/X (susceptible scion un-grafted). Ramat Negev (RN); Mivtahim (M); Amioz (A); Yated (Y) Pdaya (P); Kadesh Barnea (KB); Bet Ezra (BE).

## Discussion

ToBRFV preservation in soil could be one of the key factors contributing to recurrent viral disease in commercial tomato greenhouses (Dombrovsky et al., 2022; Klein et al., 2023; Molad et al., 2024). Micro cuts in roots while transplanting and irrigation water could promote root infection and tobamovirus transmission to above ground plant parts (Mehle et al., 2023; Zhou et al., 2024). We previously showed that ToBRFV root inoculations of tomato plants, conducted either by pouring virus sap into soil pits or by dipping truncated roots in virus sap, caused infection of the tomato plants (Dombrovsky et al., 2022; Klein et al., 2023). We also showed that ToBRFV, preserved infectious in soil following a growth cycle of infected plants, infects newly planted tomato seedlings with truncated roots (Molad et al., 2024). We have now shown soil-mediated infection of intact tomato seedlings planted in soil of infected plants from a previous growth cycle, while soil treatment with the disinfectant Cl-TSP (Dombrovsky et al., 2022; Molad et al., 2024) reduced the infection (**Figure 2A**). However, Cl-TSP disinfection did not prevent ToBRFV infection of 12.5% of the plants. Screening of leading commercial rootstocks showed that they are also susceptible to ToBRFV (**Figure 2B**), highlighting the current lack of an effective, durable strategy against soil-mediated infection.

Screening for resistant or tolerant germplasms in wild tomato species is an important strategy toward ToBRFV disease management *via* introgression of resistance genes into cultivated tomato varieties (Jaiswal et al., 2024, Jaiswal et al., 2025; Jewehan et al., 2022; Kabas et al., 2022; Rochsar et al., 2025; Topcu et al., 2025; Zinger et al., 2021). In our screening, we found two *S. pennellii* accessions: ‘LA5240’ and ‘LA716’; and one *S. chilense* accession: ‘LA1938’ as resistant toward ToBRFV showing ELISA negative results for the virus (**Table 1**). In addition, four cultivated lines harboring *Tm-1* gene were found to be resistant: ‘Rotem’ (G29884), ‘Davis_1’, ‘Davis_2’ and ‘GPT214320’ (**Table 2**). In our recent study, we mapped the resistance of *S. pennellii* ‘LA5240’ toward ToBRFV to the *Tm-1* locus on chromosome 2. A similar locus conferred resistance toward the virus in the cultivated line ‘Rotem’ (G29884) that carry the *Tm-1* introgression from *S. habrochaities* (Rochsar et al., 2025). Recent studies have confirmed *Tm-1* as the primary gene conferring resistance to ToBRFV and proposed that its effective function requires a combination with a recessive locus on chromosome 11 (Zinger et al., 2025) and an additional, as-yet-unidentified locus in the *S. pennellii* background (Zinger et al., 2025; Zois et al., 2025). This suggests that the resistance trait is more complex and is affected by multiple genetic regions across different genetic backgrounds. In this work, we evaluated more than a thousand *Tm-1* hybrids across multiple genetic backgrounds and picked the hybrids that displayed the best resistance and agronomic traits in a commercial greenhouse setting. We used the ‘Rotem’ (G29884) line to generate four resistant scions: NBK_20227, NBR_22, NBR_21784 and NBM_80, all heterozygous to *Tm-1* (**Figure 3**, **Table 2**). In addition, we used both LA5240 and Rotem (G29884) to generate two lines that served as rootstocks: one with a susceptible cultivated ‘4755’ to generate the inter specific hybrid ‘LA5240*4755’, and the second was the F_3_ line ‘33’ that derived from the ‘Rotem’ accession (**Figure 3**). Both of the rootstocks showed compatibility upon grafting (King et al., 2010) and were both resistant toward ToBRFV (**Figure 2B**).

A comparison of the *Tm-1* amino acid sequences of *S. pennellii* ‘LA5240’ (*Tm-1^LA5240^*), ‘LA716’ (*Tm-1^LA716^*), and ‘Rotem’ (G29884) (*Tm-1^Rotem^*) indicated that the resistant alleles were highly similar. *Tm-1^LA5240^* and *Tm-1^LA716^* shared 99.86% identity, while *Tm-1^Rotem^* showed a slightly lower similarity to the two wild accessions (98.85% with LA5240 and 98.94% with LA716). Their similarity to the susceptible *tm-1* allele was 88–90% (**Supplementary Figure 1**). Notably, the viral replicase-binding region, located between positions 79 and 112 within the NN-domain (Ishibashi et al., 2014), showed greater divergence. In this region, *Tm-1^LA5240^* and *Tm-1^LA716^* were identical (100%), while *Tm-1^Rotem^* shared 94.12% identity with both wild *S. pennellii* accessions. In contrast, identity to the susceptible *tm-1* allele was lower: 76.47% for ‘LA5240’ and ‘LA716’, and 70.59% for ‘Rotem’ (G29884) (**Supplementary Figure 1**). Although the resistant alleles differed slightly from one another, they showed high similarity in the binding site to the viral replication protein. The low percent identity between the resistant and susceptible alleles at this binding site may explain the functional difference between resistant and susceptible alleles, but further structural studies (e.g., X-ray crystallography) of the *Tm-1* and ToBRFV replicase protein complex are required for validation.

We now applied a genetic approach in grafted plants, to hinder soil mediated ToBRFV infections. In our results, we showed that ToBRFV root inoculations of the susceptible rootstocks ‘Rezistar’ and ‘RS-5’ caused ToBRFV plant infection when the scion was either the resistant hybrids ‘NBK_20227’ and ‘NBR_21784’ or the susceptible ‘Shiren’ cultivar (**Figure 4**, 5B). These results emphasized the possibility of soil-mediated infections of grafted plants and could infer that ‘NBK_20227’ and ‘NBR_21784’ resistance toward ToBRFV could not hinder systemic infections transmitted from the roots *via* the susceptible rootstock. Similar results were reported on systemic necrosis occurred in resistant tomato plants when TMV was introduced *via* a grafted susceptible plant, suggesting that virus introduction through the vascular system by-passes the resistance (Stobbs and Macneill, 1980). These findings could also be indicative of the difference in plant defense response toward viral infection occurring in roots compared to shoots (Andika et al., 2016). Once ToBRFV overcomes the defense response in the roots, the modified gene expression prevails over the shoot defense response. Grafting on resistant rootstocks could be beneficial to plants in their combat against ToBRFV in light of the findings that upon foliar inoculations, tobamoviruses primarily reach the roots before the systemic spread (Cheng et al., 2000; Samuel, 1934; Spanò et al., 2020). We found that upon root inoculations, the resistant rootstock ‘LA5240*4755’ conferred resistance to both the resistant scions ‘NBK_20227’ and ‘NBR_21784’ and susceptible scion ‘Shiren’ (**Figures 4**, **5B**). Upon foliar inoculations of the susceptible scion grafted on the resistant rootstock, no protection was conferred by the rootstock (**Figure 5**). However, in order to induce response to ToBRFV in the resistant rootstocks, we have root-inoculated the rootstock ‘LA5240*4755’ before foliar inoculations of the susceptible scion ‘Shiren’. We found a tendency toward low infection levels of the susceptible scion upon foliar inoculations of the combined inoculated grafted plants compared with foliar-only inoculated grafted plants. Although we applied highly stringent ToBRFV infection conditions, we could also detect in the combined inoculated grafted plants of ‘Shiren’ on ‘LA5240*4755’ a statistically significant phenotypic advantage over foliar-only inoculated grafted plants with DSI values of 55% compared to 75%, respectively (**Figure 6A**). The results of combined inoculations coincide with the finding that rootstock-specific transcripts participate in modulating graft-associated resistance toward TMV (Kappagantu et al., 2023). Importantly, in a field trial conducted during spring 2021, the resistant rootstock ‘LA5240*4755’ hindered ToBRFV disease-manifestations on fruits of the first three clusters of the grafted susceptible scion ‘Odelia’ (SS/RR), exposed to ToBRFV infection *via* commonly practiced plant manipulations such as trellising and pruning (**Figure 6C**). In addition, the susceptible rootstock ‘Beaufort’ significantly increased symptom manifestations in fruits of the first three clusters of the grafted resistant scion ‘NBR_22’ (RS/SR), when compared with the ‘NBR_22’ grafted on the resistant rootstock ‘LA5240*4755’ (RS/RR) (**Figure 6F**). These results are compatible with established literature that upon scion inoculations, TMV accumulation in a tolerant scion was higher when grafted on a susceptible rootstock compared with a scion grafted on a tolerant rootstock (Arroyo and Selman, 1977). In our field trial, similarity was observed between fruit symptom severity of the susceptible scion ‘Odelia’, grafted on the resistant rootstock ‘LA5240*4755’ (SS/RR) and that observed on the resistant scion NBR_22 grafted on the resistant rootstock ‘LA5240*4755’ (RS/RR) (**Figures 6C, F**). It is important to note that in our previous work, we showed that the *Tm-1* act in a tissue-specific manner and does not affect fruit symptom development (Rochsar et al., 2025). In the present study, we observed a significant reduction in fruit symptom severity when grafting onto the resistant rootstock ‘LA5240×4755’ compared with susceptible rootstocks. This suggests that rootstock-mediated effects may influence fruit symptom expression independently of scion resistance. This effect may reflect the action of graft-transmissible signals originating from the rootstock, consistent with recent literature in TMV showing that mobile rootstock-derived transcripts can enhance graft-associated resistance without conferring complete immunity (Kappagantu et al., 2023). Alternatively, the reduced symptom severity may result from indirect effects of the resistant rootstock on fruit quality traits, such as soluble solids content (Jenkins et al., 2022), rather than from a direct resistance mechanism.

Across seven field observations conducted between 2020 and 2023 in commercial tomato greenhouses, grafting combinations varied substantially in their response to natural ToBRFV infection. Plants grafted with resistant scions onto resistant rootstocks (RS/RR) consistently showed the lowest infection levels, whereas susceptible scions grafted onto susceptible rootstocks or grown ungrafted exhibited uniformly high infection rates (>90%) across sites and seasons. Susceptible scions grafted onto resistant rootstocks (SS/RR) displayed intermediate infection levels, indicating that resistant rootstocks can reduce, but not prevent, ToBRFV infection. In two of three field observations, SS/RR plants had significantly lower infection rates than susceptible controls, while no statistical difference was detected in the third (KB) location. Nevertheless, infection levels in SS/RR plants in KB field location did not exceed those of susceptible controls, suggesting a protective effect of the resistant rootstock that varied with field conditions. Variation among observations likely reflects differences in local infection pressure driven by the infectivity of the previous crop cycle (Molad et al., 2024), phyto-sanitation practices (Dombrovsky et al., 2022; Zamora-Macorra et al., 2025), environmental conditions (Nolasco-García et al., 2023; Oladokun et al., 2019), and repeated mechanical transmission during crop management (Ehlers et al., 2023; Panno et al., 2020; Skelton et al., 2023). Resistant rootstocks did not reduce infection following direct foliar inoculations under controlled conditions (**Figure 5**), indicating that rootstock-mediated effects do not block systemic infection once the virus enters wounded foliar tissues (Stobbs and Macneill, 1980). Instead, disease suppression under field conditions may be linked to infection routes or physiological contexts specific to commercial production systems, including soil-mediated infection (Dombrovsky et al., 2022; Klein et al., 2023; Smith and Dombrovsky, 2019), root injury during transplanting (Mehle et al., 2023; Zhang et al., 2025; Zhou et al., 2024), or graft-transmissible signaling (Kappagantu et al., 2023; Lu et al., 2020). From an applied standpoint, the reduction in infection incidence from approximately 95% in susceptible controls to 73% in SS/RR plants (**Table 3**), together with reduced fruit symptoms (**Figure 6**), observed under natural inoculation conditions, constitute a significant but partial mitigation of ToBRFV, supporting the use of resistant rootstocks as part of an integrated disease management strategy rather than a standalone solution. In contrast, the consistently low infection levels observed in RS/RR plants (16.43%) highlight the importance of combining resistance in both scion and rootstock to achieve durable control of ToBRFV under a high disease pressure. A summary of our illustrated data, presented in **Table 4**, could stress the importance of further studies focusing on the possible effects of environmental factors on mitigating ToBRFV disease. For example, temperature, humidity, and soil type could affect ToBRFV transmission *via* resistant rootstocks and provide a better understanding of how to optimize growing conditions for disease control. Resistant scions grafted onto susceptible rootstocks were ineffective against soil-mediated ToBRFV transmission, underscoring the risk of using such graft combinations in contaminated greenhouses. Hence, for agricultural implications, resistant scion grafted on resistant rootstock are necessary to ascertain the resistance trait effect upon foliar and root inoculations that occur naturally under field conditions.

**Table 4:** **A** summary of grafting strategies to mitigate ToBRFV disease.

Category	Main results	Conclusions
Rootstock Efficacy	Susceptible rootstocks mediated infection of resistant scions during root-inoculation. Conversely, resistant rootstocks (‘LA5240x4755’ and ‘33’) hindered soil-mediated infection of susceptible scions.	Grafting on resistant rootstocks provides a barrier against soil-mediated transmission but does not confer immunity to foliar-inoculated infections.
Disease & Fruit Mitigation	Field trials showed that resistant rootstocks significantly reduced fruit symptom severity (e.g., from 41.9% to 16.6% DSI) even when the scion was susceptible.	Resistant rootstocks mitigate economic damage by lowering fruit symptoms and potentially inducing systemic defense responses through graft-transmissible signals reaching mostly the first fruit cluster.
Commercial Field Performance	In large-scale field observations, the combination of resistant scions and resistant rootstocks (RS/RR) resulted in only 16.43% infection, compared to over 92% infection in susceptible controls.	For durable and sustainable disease management, integrated strategies combining both resistant scions and resistant rootstocks are necessary to combat both foliar and soil-borne infection routes

## Material and methods

### Calibrating disease symptom severity scores

Tomato plants harboring the *Tm-2^2^* resistance allele, grown in a greenhouse in Hatsav village, Israel, naturally infected by ToBRFV, were inspected over a period of 4 months to evaluate symptom intensity and severity manifested on leaves and fruits. ToBRFV symptoms were given a disease severity score between 0 (asymptomatic) and 3 (high severity). Severity scores for leaf symptoms were: 0) Asymptomatic leaves. 1) Mild mosaic leaves. 2) Severe mosaic developed on leaves, yellowing of leaf veins. 3) Narrowing of leaf tips and curling of the whole leaf. Disease severity scores for fruit symptoms were 0) Asymptomatic fruits. 1) Mature fruits have not ripened enough (blotchy ripening). 2) Yellow/green spots on fruits with necrotic lesions on peduncles, pedicels, and the calyces. 3) Brown rugose necrotic infections.

DSI (%) was calculated using the equation: {Sum (class frequency) x (score of rating class)}/{(total number of plants) x (maximal disease index)}x 100 (Chiang et al., 2017). DSI values below 20% were considered as indicative of tolerance (Bari et al., 2023; Jaiswal et al., 2024).

### Foliar and root ToBRFV inoculations

Foliar and root inoculations were conducted under stringent conditions. Foliar inoculations were conducted on the first true leaf of 30–40 days old seedlings, using sap prepared from leaves of ToBRFV-infected *Tm-2^2^* resistant tomato plants (e.g. ‘Ikram’, ‘Shiren’), (1 g leaves/10 ml 0.01M sodium phosphate buffer, pH=7.0, without carborandum). To improve viral transmission in root inoculations, the roots were manually truncated and then dipped in sap prepared as above.

### Indirect enzyme-linked immunosorbent assay

Tomato leaf samples (the second leaf beneath the meristem) were collected 30 days after mechanical inoculations of seedlings. The collected samples were subjected to an indirect ELISA test (Koenig, 1981). Each sample had a technical duplicate repeat, as well as a buffer control and positive and negative controls. Sampled leaves were ground in a coating buffer (according to Agdia protocol, Agdia, Elkhart, IN, USA) (~0.6 g/ml) and then incubated for 3 h at 37 °C for adhesion. Following x4 washes with PBS-Tween (0.05% Tween-20 in PBS), ToBRFV-specific antisera diluted 1:3000 in PBS-2% milk were added and incubated at 37 °C for 2 h. Following washes (x4) with PBS-Tween, samples were incubated with alkaline phosphatase (AP) -conjugated goat anti-rabbit (IgG) (Sigma, Steinheim, Germany) diluted 1:4000 in PBS-2% milk for 3 h at 37 °C. Following washes (x4) with PBS-Tween, 0.6 mg/mL p-nitrophenyl phosphate (Sigma) substrate was added for the detection reaction. The developed color was recorded using ELISA reader (Thermo Fisher Multiskan FC) 15–30 minutes after adding the substrate, at 405 nm and 620 nm (Clark and Adams, 1977). For quantitative trait analyses, we used the average values of the duplicates divided by the mean of healthy technical control values. Minimum ratios of more than three times the O.D. values of the negative (healthy) reference controls (NR) were considered positive for ToBRFV.

### Statistical analyses

Statistical analyses of quantitative ELISA data were performed using a General Linear Model (GLM; multifactor ANOVA), including relevant factors and their interactions. Least squares means (LSMeans) were calculated and compared using Tukey’s honestly significant difference (HSD) test at a significance level of p < 0.05. Disease symptom severity of leaves and fruits was analyzed using Student’s t-test where appropriate, followed by Tukey’s HSD test for multiple comparisons. For binary ToBRFV ELISA results (positive/negative) obtained from field observations, a Pearson chi-square test was applied. All analyses were conducted using JMP Pro 19 software (SAS Institute, Cary, NC, USA).

### Wild tomato accessions tested in a short-term glasshouse experiment

A set of 52 wild species entries from Prof. Zamir lab collection, which was originally derived from the TGRC collection in Davis California, was tested for its response to ToBRFV infection. Seeds of 52 accessions were sown in trays at ‘Hishtil’ Ashkelon (10 seeds per line). Thirty days post sowing, the young seedlings were transferred to a glasshouse in Volcani Center, and sap mechanical inoculation was then conducted. Four weeks post inoculation, leaf samples (the second leaf beneath the meristem) were collected and tested for the virus using ELISA, and the DSI was calculated for each line as well.

### Cultivated tomato lines tested in a long-term greenhouse experiment

A set of 45 lines that included cultivated commercially available lines, the experimentally selected hybrids (**Figure 2**), and inbreds from Prof. Zamir’s lab collection, was tested for susceptibility to ToBRFV, as well as 376 lines of the G2P-SOL population (www.g2p-sol.eu) that encompass the entire natural variation of tomato genes. Three of the 376 lines were symptomless and were included in our analyses (GPT200700, GPT202680, and GPT214320). Seeds of the cultivated lines and of three of the G2P-SOL population were sown in trays at ‘Hishtil’ Ashkelon (30 seeds per line). Thirty days post sowing, the young seedlings were transplanted to plots in a greenhouse in Mivtahim village, Israel (2 plots per line, 10 plants per plot). Sap mechanical inoculation of each plant was then conducted. At four weeks post-inoculation, and throughout a 4-month growing period, we evaluated the disease symptom severity on leaves and fruits, and asymptomatic lines were subjected to ELISA for ToBRFV detection.

### Amino acid sequence alignment of *Tm-1* alleles

*Tm-1* amino acid sequences were obtained from “Sol Genomics Network”, “Solanum spp. genome projects” and from the publication of Zinger et al (Zinger et al., 2025). Sequence alignment was preform using “EMBL’s European Bioinformatics Institute” web tool for multiple alignment (Madeira et al., 2024) and “BLAST” for each pair sequence identity.

### Plant grafting

Rootstock seeds were sown in 1.6-inch trays, and two days later scion seeds were sown in 1.2-inch trays at ‘Hishtil’ nursery in Ashkelon, Israel. Two weeks later the scions were grafted on the rootstocks using the tube grafting method (Lee and Oda, 2003). For the analysis of resistance of the cultivated line ‘33’, line ‘33’ served as a rootstock for the susceptible ‘Odelia’ scion (SS). For the following experiments, we used ‘LA5240*4755’ as a resistant rootstock and ‘NBK_20227’ and ‘NBR_21784’ as resistant scions (**Figure 3**). As susceptible rootstocks (SR), we used ‘RS-5’ or ‘Rezistar’, and the susceptible ‘Shiren’ cultivar served as a susceptible scion. We generated the following combinations of grafted rootstocks and scions: resistant scion/resistant rootstock (RS/RR), susceptible scion/resistant rootstock (SS/RR), resistant scion/susceptible rootstock (RS/SR), and susceptible scion/susceptible rootstock (SS/SR). We compared self-grafting with non-grafted susceptible scions to test the effect of grafting itself on response to ToBRFV inoculations. We then used non-grafted scions as controls. Two experiments with the grafted plants were conducted during two seasons, spring and fall, in a net house at Volcani Center where the plants (10 plants for each treatment/grafted combination) were exposed to ToBRFV infection *via* either foliar or root inoculations.

Between 2020 and 2023, 6 field observations of grafted plants were conducted in commercial greenhouses where the plants were subjected to ToBRFV infection through routine plant manipulations, including trellising and pruning, as well as soil-mediated infections. Plant leaves were then tested for the virus using ELISA. The following grafting combinations were evaluated: RS/RR, SS/SR, and SS/RR. The resistant scions used were ‘NBK_20227’, ‘NBR_21784’, NBR_22, and ‘NBM_80’, and the resistant rootstock was ‘LA5240*4755’ (**Figure 3**). The susceptible scions included ‘Shiren’, ‘Ikram’, and ‘Torry’, and the susceptible rootstocks were ‘Beaufort’, ‘RS-5’, and ‘Kardia’. Each field trial consisted of 70–160 grafted plants per each combination, and random samples (ranging from 10 to 80 samples per a grafting combination) were collected for ELISA test during the mid-to-late stages of the growing season.

In addition, in one experiment conducted in a commercial greenhouse, where the grafted plants were similarly subjected to ToBRFV infection *via* regular plant manipulations, as well as soil-mediated infections, the plants were grown for 4 months to the fruiting stage and scoring of the leaf and fruit phenotype was done for each cluster. The following combinations of grafted plants were tested: RS/RR, RS/SR, SS/SR, and SS/RR. The study was conducted on 2 lines of 7–10 plants each, for each of the grafted combinations. The resistant scion was ‘NBR_22’, and the resistant rootstock was ‘LA5240*4755’ (**Figure 3**). The susceptible scion was ‘Odelia’, and the susceptible rootstock was ‘Beaufort’.

### Soil-mediated ToBRFV infection of tomato plants grown on soil of a previous growing cycle of infected crops

To study soil-mediated ToBRFV infection under regular planting procedures, *Tm-2^2^*-resistant tomato seedlings, at a 2–4 true leaf stage, were planted in an R&D greenhouse in Southern Israel following a growth cycle of ToBRFV-infected crops. The experiment was conducted with 384 ‘Ikram’ plants divided into two groups: one group was planted regularly, and the second group was planted in 3% Cl-TSP-treated soil. The Cl-TSP was poured into the planting pit before placing the seedlings. At 21 days post planting plant leaves were sampled for ELISA test.

### Western blot analyses

Sampled leaves were weighed and subjected to protein extraction by crushing leaves in USB buffer containing 75 mM Tris-HCL (pH6.8), 8 M urea, 4.5% (*g/v*) SDS, and 7.5% (*v/v*) ß-mercaptoethanol, keeping a constant ratio of 1 µg leaf/5.5 µl USB buffer. The samples were then incubated at 96 °C for 15 min and micro-centrifuged at 16,000 *g* at 4 °C for 15 min. The supernatant was then mixed with Laemmli buffer (Laemmli, 1970) and run on 12.5% SDS-PAGE. The separated proteins were electro-blotted onto a nitrocellulose membrane at 200 mA for 36 min (for one gel), and the membranes were then incubated at room temperature for 2 h in PBS containing 3% non-fat dry milk for blocking. After blocking, the membranes were subjected to ToBRFV detection by incubation overnight at 4 °C with specific antibodies raised against ToBRFV coat protein (CP) (Luria et al., 2017) diluted 1:4000 in PBS. For detection of ToBRFV CP, the membranes were first washed with PBS-Tween buffer and then incubated with alkaline phosphatase (AP) conjugated goat anti-rabbit antibodies (Sigma, Steinheim, Germany) for an hour at room temperature using 1:5000 dilution in PBS. Following washes with PBS-Tween, ToBRFV CP was detected by the addition of an AP-substrate NBT, BCIP (Bio-Rad, CA, USA). Following CP detection Ponceau-s staining was conducted using 0.2% (*w/v*) Ponceau-s in PBS containing 5% acetic acid.

### DNA extraction and genotyping using CAPS markers

Genomic DNA was extracted from young tomato leaves (~15 mg per sample) following the protocol of Eshed and Zamir (1995) (Eshed and Zamir, 1995). The wild and cultivated species mode of inheritance of *Tm-1* was genotyped and confirmed using Cleaved Amplified Polymorphic Sequence (CAPS) analysis. Marker design was performed using SnapGene (GSL Biotech, USA) based on sequence information obtained from the Sol Genomics Network (SGN). PCR amplification was performed using the primers 5′-TGCTGCACCCGCATTAGATAG-3′ (forward) and 5′-GTGACAGTTGTTGATGTCTCAACC-3′ (reverse) in a final reaction volume of 13 µL, containing 3 µL genomic DNA and 10 µL of a master mix composed of Taq DNA polymerase mix, primers, and nuclease-free water. Cycling conditions included an initial denaturation at 95 °C for 1 min; 30 cycles of 95 °C for 30 s, 55 °C for 15 s, and 72 °C for 15 s; followed by a final extension at 72 °C for 5 min. PCR products were cleaved with HaeIII in CutSmart buffer at 37 °C for 1 h and separated on a 2.5% agarose gel to determine allele identity. Cultivated *tm-1* genotypes displayed fragments of 208, 137, and 84 bp, whereas homozygous wild *Tm-1* plants showed fragments of 137, 112, 96, and 84 bp. Heterozygous individuals exhibited a combination of both patterns. During the generation of experimental hybrids, the *Tm-1* genotype was determined at each breeding stage using both the CAPS marker described above and the *Tm1*_Rotem marker previously reported by (Rochsar et al., 2025). The *Tm1*_Rotem marker is specific to the *Tm-1 Rotem* allele and was used to confirm its presence in the breeding lines. In addition, it was used in combination with the CAPS marker to rule out or verify the presence of the *Tm-1 LA5240* allele by comparing the two marker profiles, ensuring accurate allele identification in all breeding stages.

## Data Availability

The original contributions presented in the study are included in the article/**Supplementary Material**. Further inquiries can be directed to the corresponding authors.
