# Supplementary material for: Breeding Tm-1-based tomato rootstocks, resistant to tomato brown rugose fruit virus, to impede soil-mediated viral infections
Source: Front Plant Sci. 2026 Apr 23;17:1815342. doi: 10.3389/fpls.2026.1815342 (PMC13149247; doi:10.3389/fpls.2026.1815342)
Supplement: Supplementary file 1 [file Table1.docx]

**Supplementary Figure S1**

**Figure S1**- amino acid sequence comparison between the resistant LA5240 (*Tm-1^LA5240^*), LA716 (*Tm-1^LA716^*), Rotem (G29884) (*Tm-1^Rotem^*) and the susceptible culativated *tm-1* alleles. The area marked in yellow between amino acid 79-112 is the binding site of the *Tm-1* protein to the viral replication protein.
